# Evaluation of Graphene/WO_3_ and Graphene/CeO_*x*_ Structures as Electrodes for Supercapacitor Applications

**DOI:** 10.1186/s11671-017-2385-1

**Published:** 2017-12-22

**Authors:** Stefanos Chaitoglou, Roger Amade, Enric Bertran

**Affiliations:** 1 FEMAN Group, Department of Applied Physics, Universitat de Barcelona, C/ Martí i Franquès, 1, 08028 Barcelona, Spain; 2 Institute of Nanoscience and Nanotechnology (IN2UB), Universitat de Barcelona, Barcelona, Spain; 3 Institute of Nanoscience and Nanotechnology, NCSR DEMOKRITOS, 15310 Aghia Paraskevi, Athens Greece

**Keywords:** Supercapacitors, Graphene hybrid electrodes, Chemical vapor deposition

## Abstract

The combination of graphene with transition metal oxides can result in very promising hybrid materials for use in energy storage applications thanks to its intriguing properties, i.e., highly tunable surface area, outstanding electrical conductivity, good chemical stability, and excellent mechanical behavior. In the present work, we evaluate the performance of graphene/metal oxide (WO_3_ and CeO_*x*_) layered structures as potential electrodes in supercapacitor applications. Graphene layers were grown by chemical vapor deposition (CVD) on copper substrates. Single and layer-by-layer graphene stacks were fabricated combining graphene transfer techniques and metal oxides grown by magnetron sputtering. The electrochemical properties of the samples were analyzed and the results suggest an improvement in the performance of the device with the increase in the number of graphene layers. Furthermore, deposition of transition metal oxides within the stack of graphene layers further improves the areal capacitance of the device up to 4.55 mF/cm^2^, for the case of a three-layer stack. Such high values are interpreted as a result of the copper oxide grown between the copper substrate and the graphene layer. The electrodes present good stability for the first 850 cycles before degradation.

**Electronic supplementary material:**

The online version of this article (10.1186/s11671-017-2385-1) contains supplementary material, which is available to authorized users.

## Background

Recently, electrochemical energy storage devices such as supercapacitors are becoming the most popular apparatus as power supplies in a wide variety of applications, from portable electronic devices, such as cell phones and laptops, to hybrid electric vehicles [1]. Supercapacitors can exhibit a higher power density and a superior cycle life when compared to conventional batteries. At the same time, they exhibit a lower energy density [2].

This is a result of the different energy storage mechanism between the two devices. In contrast to batteries, where ions are stored through chemical bonding to the electrode materials, in supercapacitors, an electrostatic storage of the energy takes place through the separation of charge in a Helmholtz double layer [3]. Additionally, supercapacitors exhibit pseudocapacitance through surface redox reactions that contribute as electrochemical energy storage. The storage mechanism here is based on faradaic redox reactions with charge-transfer. Various metal oxide materials are investigated for this purpose, as the energy density associated to faradic redox reaction is an order of magnitude higher than that attributed to double-layer capacitance.

Hence, it is considered that supercapacitors have the potential to replace or complement batteries in energy storage applications. Research in this direction focuses in the development of novel electrodes that can exhibit superior characteristics. Similar to Li ion batteries, carbon-based materials are preferred due to their low environmental impact, chemical stability, high conductivity, and low cost [4].

Graphene, an emerging nanomaterial which consists of all sp^2^-hybridized carbon atoms, has some very exciting properties which make it very attractive for being used as an electrode in this kind of applications. We highlight its light weight, high electrical and thermal conductivity, highly tunable surface area (up to 2675 m^2^/g), strong mechanical strength (~ 1 TPa), and chemical stability [5–7]. Single-layer graphene exhibits a theoretical specific capacitance of around 21 μF/cm^2^ and a corresponding specific capacitance of around 550 F/g when the entire surface area is fully utilized. At present, it is given much attention to three-dimensional graphene materials, such as graphene nanowalls and nanofoams, which can deliver high energy density and power density, in the order of 13 Wh kg^−1^and 8 kW kg^−1^, respectively [8]. However, these materials need a more complex plasma-enhanced growth technology, to increase the plasma density, which makes difficult the control of homogeneity [9].

Further, planar graphene films present the benefit of a homogeneous growth and good coupling to the metallic substrate, resultant of the mixture of covalent and ionic bonding on the graphene/copper interface [10], which serves as current collector. However, planar single-layer graphene film has a relatively small surface area which does not promote the storage of high amounts of energy. A popular approach to overcome this is to combine graphene with other materials that can store energy.

The recent advances in the design and optimization of higher efficiency electrodes has promoted the combination of graphene and graphene oxide films with different metal and metal oxide composites [11–19], like metal oxide nanoparticles, to build hybrid supercapacitors. Such metal oxide structures contribute to the total capacitance by providing a high pseudocapacitance due to faradic redox reactions taking place on large surface area electrodes.

On this design, graphene contributes, apart from its storage capacity, as a platform which permits the strong coupling and a good electric contact between the metallic nanoparticles and the current collector. Previous studies have revealed the beneficial role of graphene as a coupler between the current collector and carbon nanotubes [20].

On other research work, single-layer graphene electrodes have been measured to exhibit a specific double-layer capacitance of up to 135 F/g, while when combined with other compounds such as Fe_2_O_3_ and MnO_2_, they show capacitances up to 380 F/g [21, 22].

In the present work, we have manufactured graphene/metal oxide nanocomposites made from a single layer or three stacked layers of graphene/metal oxide nanocomposites, combining graphene transfer and magnetron sputtering techniques. On top of each graphene layer, different metal oxide particles of WO_3_ and CeO_*x*_ were sputtered.

Cerium oxide is mentioned as CeO_*x*_ in the whole manuscript as we have not characterized the grown particles. Although the sputtering process was performed with a CeO_2_ target, the formed particles should appear suboxidized because of the possible loss of oxygen during the sputtering process, but they are mainly formed by CeO_2_, which is the most stable form of cerium oxide. Compared to monolayer graphene, stacks of graphene films have more electrode/electrolyte interfaces, which is beneficial to the absorption/desorption of electrolyte ions and provide more electrical pathways for electrolyte ions during charging and discharging processes. The deposition of metal oxide particles enhances the specific capacitance of ultrathin layers at a relatively low mass loading [23]. In previous works, CeO_*x*_ particles have demonstrated high capacitance, in the order of 119 mF/cm^2^, when combined with nickel foam [24]. Considering the WO_3_ films, a recent work has reported electrodes made with WO_3_ rods presenting a capacitance of 266 F/g [25]. Both nanocomposites have shown favorable electrochemical redox characteristics and ion reactivity. We have chosen the above metal oxides since we did not find any recent work reporting their combination with chemical vapor deposition (CVD)-grown graphene films. Thus, we proceed to study how these hybrid composites combine between each other and the capacitance characteristics of the resulting electrodes.

The use of the same experimental conditions in the preparation of the two hybrid materials provides us with the opportunity to directly compare the electrochemical performance of the electrodes.

To better interpret our results, we take into consideration the contribution of the native copper oxide layer in the overall capacitance of the electrode.

## Experimental

### Hybrid Electrode Preparation

**Fig. 1:**
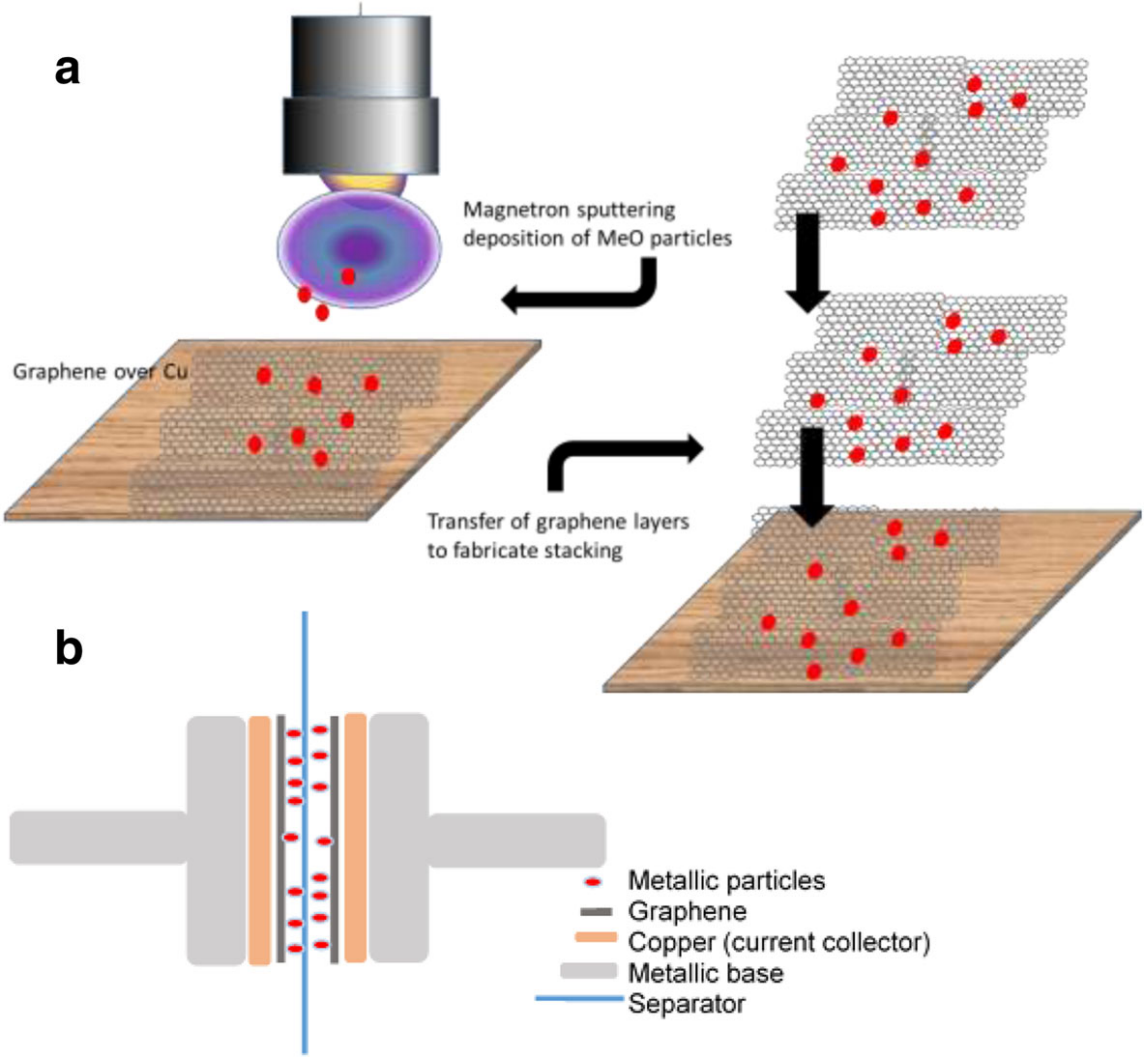
Schematic drawings. Detailed legend: **a** Schematic drawing showing the preparation process of the graphene/MeO stacking. **b** Scheme of the design of the cell. The separator (glass fiber filter) is soaked with 1 M LiClO4 dissolved in ethylene carbonate (EC) and diethyl carbonate (DEC) mixed in 1:1 volumetric proportions

Continuous graphene films were grown by CVD following the growth recipes described in our previous work [26]. We report briefly the growth process. Polycrystalline copper foil (75 μm thick, 99% pure) was cut in ~ 0.7–1.0 cm^2^ pieces, cleaned in ultrasound bath of isopropanol and acetone, 10 min each, to remove impurities and loaded in the chamber. First, we apply a hydrogen plasma etching to remove the native copper oxide from the copper surface. Radio frequency (RF) plasma is generated by applying 100 W at 20 Pa pressure, under 20 sccm hydrogen flow. The plasma etching lasts 10 min. Then, the sample is forwarded in a quartz tube (coupled to the plasma chamber) surrounded by an oven. The oven is heated at 1040 °C and the gases are introduced in the tube. A mixture of methane and hydrogen is introduced (5/20 sccm of methane/hydrogen) during 20 min at 15 Pa, resulting in the complete coverage of the copper foil by single-layer graphene. Then, the sample is let to cool down in room temperature in high vacuum (3 × 10^−4^ Pa) before being removed from the chamber. Then, the sample is placed in another reactor to deposit the metal oxide particles. The metal oxide particles were deposited on the graphene layer by pulsed reactive magnetron sputtering (1 Pa, 13/7 sccm/sccm of Ar/O_2_ flow, 60 W, 5 s of deposition time, target-substrate distance 10 cm), using, each time, the corresponding target (W or Ce). To prepare three stacked layers of graphene/metal oxide nanocomposites, we used graphene transfer method with a polymer coating support [26]. Polymethyl-methacrylate (PMMA) is spin-coated over the graphene, and then the sample is immersed into FeCl_3_ to etch away the copper. The remaining graphene/metal oxide layer was then transferred over another layer of the same nanocomposite enabling the preparation of the stacked material. After the transfer process, the PMMA was removed by rinsing with acetone. The preparation process of the composite is presented in the schematic drawing of Fig. 1a.

### Structural/Morphological Characterization

Samples were characterized by Raman spectroscopy (Jobin-Yvon LabRam HR 800), scanning electron microscopy (SEM) (JEOL JSM7100F), and transmission electron microscopy (TEM) (Bioscan Gatan JEOL 1010). X-ray photoelectron spectroscopy (XPS) measurements were performed in a PHI 5500 Multitechnique System (from Physical Electronics) with a monochromatic X-ray source (Al K_α_ line of 1486.6 eV energy and 350 W). Depth profile measurements of chemical composition by XPS were obtained by sputtering the surface with an Ar^+^ ion source (4 keV energy). All these measurements were made at ultra high vacuum (UHV) conditions, between 7 × 10^−7^ and 3 × 10^−6^ Pa.

### Electrochemical Characterization

The electrochemical properties of the samples were analyzed using a Swagelok cell and organic (1 M LiClO_4_ solved in ethylene carbonate (EC) and diethyl-carbonate (DEC) mixed in 1:1 volumetric proportions) electrolytes. A glass fiber filter served as separator (Whatman glassy-fiber GF/A). Figure 1b shows a schematic drawing of the as-used cell (with one layer of graphene/metal oxide particles on each electrode) that was used for the electrochemical characterization measurements. The cell was fabricated in a MBRAUN Unilab dry N_2_ glove box (< 1 ppm O_2_ and < 1 ppm H_2_O) by sandwiching an organic electrolyte-soaked separator between two graphene/MeO electrodes. To study the supercapacitance behavior of the devices, we first performed cyclic voltammetry (CV) measurements at different scan rates and with a voltage window of 1.8 V.

## Results and Discussion

### Hybrid Structure

**Fig. 2:**
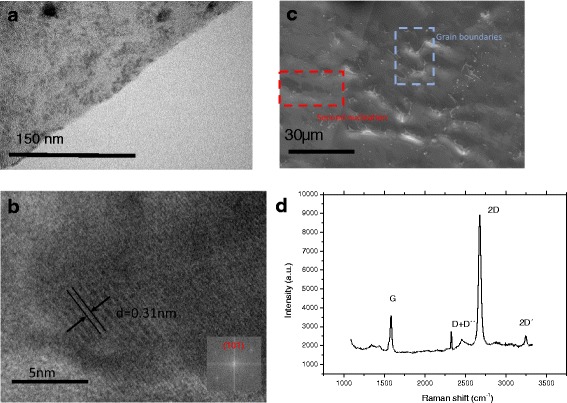
Morphological and structural characterization. Detailed legend: **a** TEM image of Gr/WO3 film structure and Raman spectra. **b** HRTEM image of Gr/WO3 and diffraction pattern of tungsten oxide corresponding to standard tetragonal (101) of WO3. **c** SEM image of the as-grown continuous graphene film. **d** Raman spectrum of the as-grown graphene film after transferring over SiO2

The short sputtering deposition of the MeO nanoparticles aims in avoiding the damage of the graphene layer. Longer sputtering periods could result in the damaging of the graphene, since the sputtering is performed in an argon/oxygen plasma. Figure 2a, b shows the TEM images of the tungsten oxide particles deposited on the graphene layer. Figure 2a shows the edge of the graphene film decorated with homogeneously distributed particles on the up-left part of the image. The larger particles have a diameter of 25 nm. Figure 2b provides a high-resolution TEM image of some larger tungsten oxide particles. The d-spacing of the particle is measured 0.31 nm, as confirmed by the selected area electron diffraction (SAED) pattern (inset Fig. 2b), corresponding to a standard tetragonal system (101) of WO_3_. The SEM images provide information about the continuity of the graphene film (Fig. 2c). We observe that all area is covered with single-layer graphene. Despite some visible grain boundaries (contained in the blue square), most graphene grains have reached the coalescence phase, forming a continuous layer. Some regions with a darker contrast (contained in the square) are the result of the nucleation of a second graphene layer, although these regions are a very small percentage of the total area, as we observe in the image. By evaluating the information provided by the Raman spectrum (Fig. 2d), the *I*_2D_ */I*_G_ intensity ratio (~ 2.47) and the 2D peak FWHM (~ 40 cm^−1^) confirm that graphene is a single layer. The spectrum was obtained after transferring the graphene film over a SiO_2_ substrate in order to eliminate the noise resulting from the copper foil luminescence [27].

**Fig. 3:**
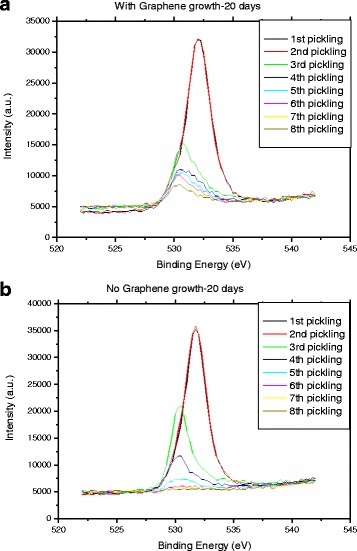
XPS characterization. Detailed legend: XPS curves with the O1s spectrum for the polycrystalline copper surface measured after various consecutive annealing processes **a** with graphene grown on top and **b** without graphene grown on top

XPS provided information considering the copper oxide formation after plasma annealing and graphene growth. The measurements were performed on copper substrates with and without graphene grown on top in order to show that the graphene presence favors the formation of the copper oxide layer. The native copper oxide layer was reduced by plasma annealing in all samples (see also the “Experimental” section), with and without graphene. We perform pickling of the surface to observe the changes in its composition. Figure 3a, b shows the O1s spectra of polycrystalline copper surface in a substrate with graphene grown on top and without graphene growth, respectively. Both samples were annealed to remove native copper oxide 20 days before the XPS measurement. The different spectra in each figure correspond to the measurements made immediately after the sample annealing processes. (see the “Experimental” section).

To obtain information about the amount of oxygen in the copper, we compare the intensities of the peaks. We study the intensity ratio between peaks with respect to the first measurement (black line). After each pickling process, we obtain information on the chemical composition at the most depth. The first two spectra (black and red line) have the same intensity. The rest of the spectra have a lower intensity. Defining the *I*_*n*_ */I*_1_ ratio*,* where *I*_*n*_ is the peak intensity of the *n* spectra and *I*_1_ the peak intensity of the first spectra, obtained by surface measurement, from Fig. 3, *I*_*n*_ */I*_*1*_ O1s ratio decreases with the increase of *n*. Although, for same *n*, the ratio is higher in the sample with graphene, revealing a higher concentration of oxygen (see Table 1 for additional information) and therefore a thicker copper oxide layer; we should underline that we do not have information about the thickness of the layer which is removed after each pickling process. The calibration is performed on a SiO_2_ film and results in a ~ 5-nm removal after each pickling. Thanks to the above XPS analysis, we conclude that oxygen is always present in the copper foil, on the naked copper, and also under the graphene layer. Also, we obtain information about the increase in depth oxidation of the copper when graphene is grown on top. Copper oxide is contributing with its capacitance to the overall capacitance of the electrode.

**Table 1:** Information about the intensity of the O1s spectrum peak with and without graphene after various annealing steps, as extracted from Fig. 5

Number	*I*_*n*_(a.u.) with graphene	*I*_*n*_(a.u.) without graphene	*I*_*n*_/I_1_ ratio with/without graphene
1	32,225	35,850	–/–
2	32,020	35,195	0.99/0.98
3	15,290	21,045	0.47/0.58
4	10,995	11,720	0.34/0.32
5	10,565	7455	0.32/0.20
6	9490	5850	0.29/0.16
7	9650	5750	0.29/0.15
8	8650	5750	0.26/0.15

### Electrochemical Results

**Fig. 4:**
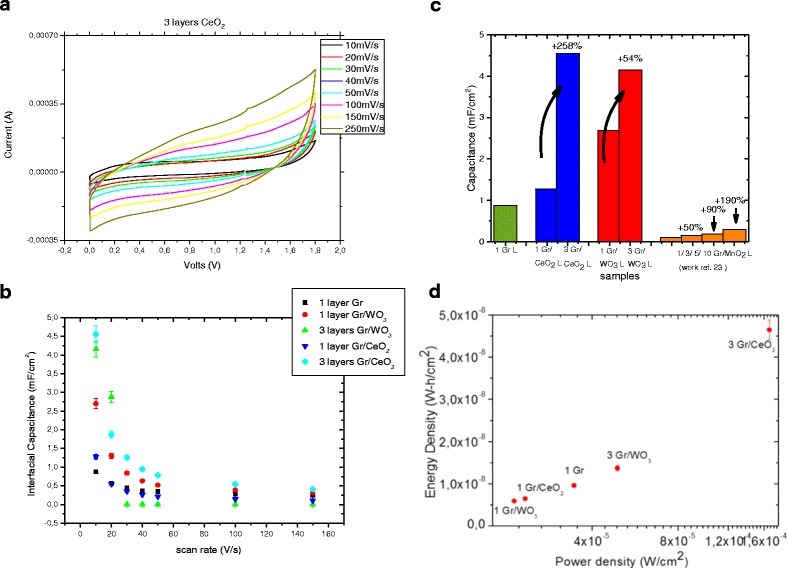
Electrochemical characterization. Detailed legend: **a** CV measurements of the cell consisted of electrodes with three layers of graphene/CeO_2_ particles each, at different scan rates. **b** Interfacial capacitance of the different hybrid electrodes at different scan rates. All devices present the higher capacitance at the lower scan rates. **c** Histogram with the percentage increase of capacitance with respect to number of layers. **d** Ragone plot demonstrating the overall performance of the graphene-based supercapacitors

In Fig. 4a, we present the CV measurements of the three layers of graphene/CeO_*x*_. The specific capacitance, *C*_s_, was calculated by the equation,

$$ {C}_{\mathrm{s}}=\frac{q_{\mathrm{a}}+\mid {q}_{\mathrm{c}}\mid }{2m\Delta V} $$

where *C*_s_ is the specific capacitance in farad per gram, *m* is the mass of the active material in grams, Δ*V* is the voltage window in volts, and *q*_a_ and *q*_c_ are the anodic and cathodic charges in coulomb, respectively.

The interfacial capacitance, *C*_i_, was calculated using the relation,

$$ {C}_{\mathrm{i}}=\frac{C_{\mathrm{s}}}{A} $$

where *A* is the area of active material dipped in the electrolyte (Fig. 4b).

The as-grown graphene film presents an interfacial capacitance *C*_i_ of 0.87 mF/cm^2^ at 10 mV/s scan rate. The capacitance decreases with the increase in the scan rate for all electrodes. The addition of MeO particles results in an increase of the electrode capacitance. Graphene films sputtered with WO_3_ particles present a capacitance of 2.69 mF/cm^2^ at 10 mV/s of scan rate and those sputtered with CeO_2_ particles a capacitance of 1.27 mF/cm^2^ at the same scan rate. The increase in the number of layers increases slightly the capacitance of the devices. Specifically, the electrodes consisting of one layer of Gr/CeO_*x*_ have a capacitance of 1.27 mF/cm^2^, which increases up to 4.55 mF/cm^2^ when two more layers of Gr/CeO_2_ are added (+ 258%). A similar behavior, resulting although in a smaller capacitance increase, is observed for the Gr/WO_3_ electrodes. Their capacity increases from 2.69 to 4.15 mF/cm^2^ when two more layers of Gr/WO_3_ are added over the first layer (+ 54%).

Similar percentage increase is expected when more graphene/metal oxide layers are added, as the surface area will increase proportionally while the interlayer distance may also allow multilayer ion absorption. In Fig. 4c, we present a histogram with the percentage evolution of the electrode capacity when more layers are added. We also include the percentage increase from Ref. 23 where a similar system with up to 10 layers is studied. Our results, considering Gr/WO_3_ electrodes, reveal an agreement in the percentage increase with respect to the Gr/MnO_2_ hybrid structure.

To demonstrate the overall performance of the supercapacitors, we illustrate a Ragone plot with the energy density and power density of the various electrodes (Fig. 4d). We observe that with the increase in the number of layers, the power density increases, reaching values in the order of 1.6 × 10^−4^ W/cm^2^ in the case of three layers of Gr/CeO_*x*_ electrodes, a value of the same order of magnitude as that of other electrodes, with similar architecture, combining graphene with MnO_2_ particles [23]. Although our device does not present a comparable energy density to the one of the above publication, in the present study, the power density has a maximum value of 4.5 × 10^−8^ W-h/cm^2^, a value which is two orders of magnitude lower than the values given for the case of Gr/MnO_2_-based electrodes.

**Fig. 5:**
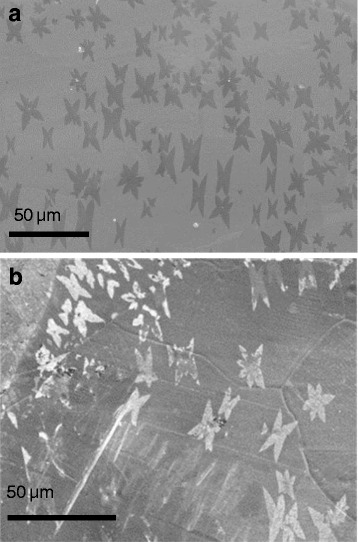
SEM characterization. Detailed legend: SEM images of **a** of the grown graphene on top of the copper catalyst before the delamination process and **b** Cu2O domains reproducing graphene “fingerprints,” as a result of the copper oxide formation

We observe that the capacitance of the sample with single-layer graphene is much higher, about nine times, than those mentioned elsewhere [23]. In the work of Zang X. et al., surface capacitance of single-layer graphene electrodes is measured to be 0.10 mF/cm^2^, while in our work, it is measured to be 0.87 mF/cm^2^. In our work, the graphene layer was deposited on a copper foil, which was used as a current collector, making transfer of graphene unnecessary. We consider that the formation of copper oxides in the graphene/copper interface, resulting from the copper oxidation, affects the total capacitance of the system. Additionally, we know that the presence of graphene favors the growth of a copper oxide layer of some tenths of nanometer, as it has been observed by us and also reported by other authors [28, 29]. Although graphene is considered an efficient oxidation barrier for Cu on a short time scale (minutes to hours), it appears to promote the galvanic corrosion of it at ambient temperature over a longer time scale [28]. By delaminating graphene from the copper surface through an electrochemical process, we can return to observe the copper substrate. Through the SEM exploration of the copper surface, a higher copper oxide formation was observed just in the areas of the foil that were covered with graphene (for more details, see Additional file 1 considering the electrochemical delamination process). Figure 5 shows the SEM images of the copper surface with graphene crystals grown over it (Fig. 5a) and after the delamination of the graphene (Fig. 5b). Bright fingerprints that reproduce the shape of the graphene domains are most probably copper oxide (Cu_2_O) layers. Their “brighter” appearance is the result of the higher backscattering of electrons on copper oxide than in the case of naked copper.

Therefore, to better interpret our results, we should consider that each electrode consists of two capacitors, the graphene film and the copper oxide film, in series, contributing to the total capacitance, as

$$ \frac{1}{c_{\mathrm{t}}}=\frac{1}{c_{\mathrm{ox}}}+\frac{1}{c_{\mathrm{g}}} $$

where *c*_t_ is the total capacitance that we measure, *c*_ox_ the capacitance of the copper oxide, and *c*_g_ the graphene quantum capacitance. Although, as it has been evaluated by experimental observations, graphene presents a negative capacitance when it is decorated with metallic adatoms. These adatoms act as resonant impurities and form nearly dispersionless resonant impurity bands near the charge neutrality point (CNP). Resonant impurities quench the kinetic energy and drive the electrons to the regime dominated by the Coulomb energy with negative compressibility. If we consider a negative quantum capacitance of the graphene [30] with a value of Ref. [23] (0.1 mF/cm^2^), we will be able calculate the copper oxide capacitance (11.1 mF/cm^2^) that corresponds to a copper oxide thickness of approximately tenths of a nanometer [31], in agreement with the experimental observation by Schriver et al. [28], considering the formation of copper oxide.

**Fig. 6:**
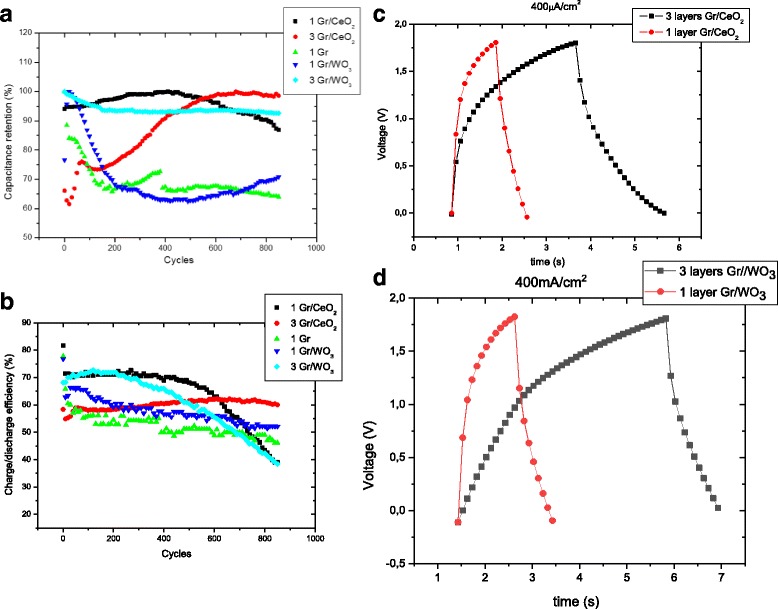
Electrode efficiency. Detailed legend: **a** Capacitance retention of the different electrodes and **b** charge/discharge efficiency. **c** Charge-discharge cycle of one and three layers of Gr/CeO_*x*_. **d** Similar for the Gr/WO_3_ hybrid

Finally, we present results considering the stability in the device performance. All electrodes present a capacitance retention of between 70 and 90% during the first 850 cycles, as we can see in Fig. 6a. According to the results of Liu et al. [32], the principal decay in the capacitance during the first cycles can be attributed to the pulverization of original metal oxide and in situ formed metal nanoparticles during Li insertion and extraction process, which leads to a loss of electrical connectivity between neighboring particles, such as we have observed in the cases of one-layer Gr/WO_3_ and three-layer Gr/CeO_*x*_. The electrodes consisting of Gr/CeO_*x*_ have a better charge/discharge efficiency during more cycles as shown in Fig. 6b. The performance of all devices is between 60 and 70%.

The galvanostatic charge/discharge curves reveal that when more layers of metal oxide/graphene are added, more time is needed for the charging and discharging process. This is visualized in Fig. 6c for the Gr/CeO_*x*_ hybrid and in Fig. 6d for Gr/WO_3_ hybrid. One-layer Gr/CeO_*x*_ needs 1.7 s approximately for a charge/discharge cycle when charged by 400 mA/cm^2^. When two more layers were added over the first one, this period increased to ~ 4.7 s. Measurements performed on a single-layer graphene showed similar charge/discharge time as in the case of the single-layer Gr/CeO_*x*_ electrodes. Similar results were obtained in the case of WO_3_ particles, where charge-discharge time was 1.9 s for one layer and 5.5 s for three layers. This demonstrates the higher power density that the CeO_*x*_ hybrid is performing. The electrochemical results of the study are listed in Table 2.

**Table 2:** Results from the electrochemical characterization of the different hybrid electrodes

Sample	Capacitance (mF/cm^2^)	Capacitance retention (1000 cycles)	Charge/discharge efficiency (%, after 900 cycles)	Charge/discharge time (s)
1-layer Gr	0.87	–	–	1.7
1-layer Gr/CeO_2_	1.27	90	38	1.7
3-layer Gr/CeO_2_	4.55	98	60	4.7
1-layer Gr/WO_3_	2.69	48	50	1.9
3-layer Gr/WO_3_	4.15	95	40	5.5

## Conclusions

Layer-by-layer evaluation of graphene electrodes combined with different metal oxides has been performed. The deposition of metal oxide particles over the graphene increases the total capacitance of the hybrid material, as metal oxide particles contribute with an additional pseudocapacitance. An increase has been also observed when more layers of Gr/metal oxide were added over the first layer. The devices in which Gr is combined with CeO_*x*_ have a slightly higher charge/discharge efficiency than those in which Gr is combined with WO_3_. Considering stability, all devices maintain their initial performance for more than 800 cycles. The charge/discharge period increases about 2.5 times with the addition of two more layers over the first one.

## Additional file

Additional file 1: Figure S1. Setup of the cell for the electrochemical delamination transfer processes. (DOCX 46 kb)
