# Supplementary material for: Evaluation of Graphene/WO3 and Graphene/CeOx Structures as Electrodes for Supercapacitor Applications
Source: Nanoscale Res Lett. 2017 Dec 22;12:635. doi: 10.1186/s11671-017-2385-1 (PMC5741570; doi:10.1186/s11671-017-2385-1)
Supplement: Additional file 1: Figure S1. — Setup of the cell for the electrochemical delamination transfer processes. (DOCX 46 kb) [file 11671_2017_2385_MOESM1_ESM.docx]

Evaluation of graphene/WO_3_ and graphene/CeO_2_ structures as electrodes for supercapacitor applications.

Stefanos Chaitoglou^1,2,3^, Roger Amade^1,2^ and Enric Bertran^1,2^

^1^FEMAN Group, Department of Applied Physics, Universitat de Barcelona,

^2^Institute of Nanoscience and Nanotechnology (IN2UB), Universitat de Barcelona

^3^ Institute of Nanoscience and Nanotechnology, NCSR DEMOKRITOS, 15310 Aghia Paraskevi, Athens, Greece

C/ Martí i Franquès, 1, 08028, Barcelona, Spain.

*Corresponding author: ebertran@ub.edu

Tel. 0034 934021135

**Supplementary Information**

We perform electrochemical delamination of graphene films from the copper substrate. This provides us with the opportunity to reuse the copper foil for further graphene growths as well as observe the condition of the copper surface after the graphene delamination. We perform the delamination following a technique similar to Wang et al^1^.

In a delamination process PMMA is spin coated on top of the CVD grown graphene previously grown on top of copper foil. The role of the PMMA here is to protect/support the layer of graphene during the delamination. Then, the stacking is introduced in an aqueous solution of potassium persulfate (K_2_S_2_O_8_) and the electrochemical process can be performed.

The copper/graphene/PMMA is the cathode electrode and glassy carbon is used as the anode electrode. As the graphene/copper electrode is cathodically polarized, hydrogen bubbles are emerging in the copper-graphene interface due to the water reduction. These bubbles are applying a small but continuous force to the graphene until the moment it detaches from the catalyst foil. The role of PMMA here is crucial. The bubbles apply forces that tend to scroll the graphene sheet instantly once it is released. The PMMA coat acts as a support for the graphene sheet preventing it from shearing and scrolling.

The aqueous solution used for the delamination was K2S2O8 of 0.5 mM concentration. The applied voltage is 10V. A scheme of the setup is presented in Figure 1.


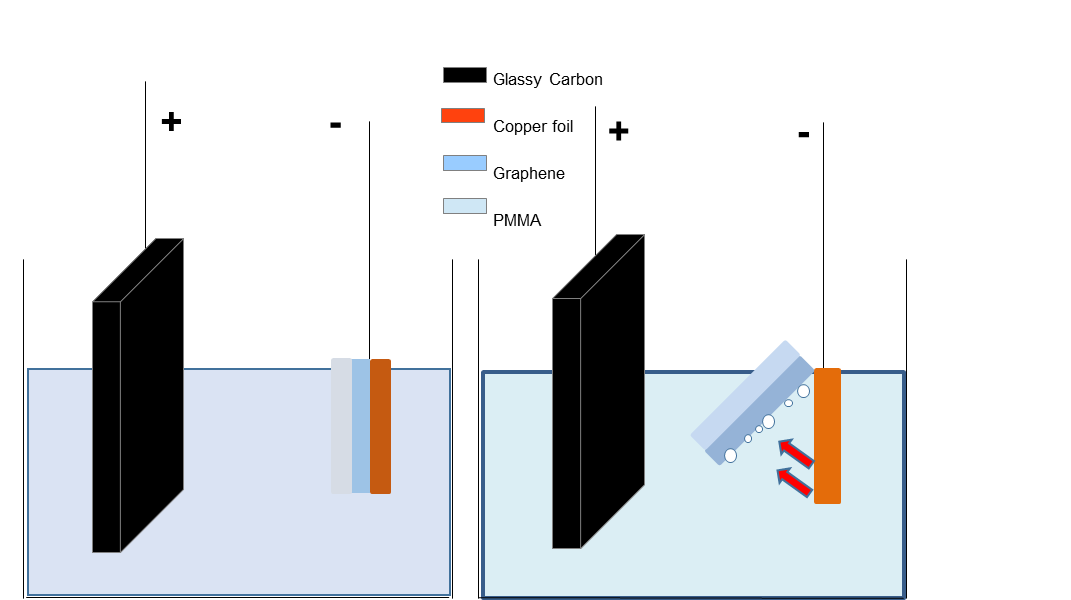


Figure 1: Set up of the cell for the electrochemical delamination transfer processes. In the left part we see the geometry of the system in the beginning of the process. In the right part we see the final stage of the process, when the graphene +PMMA have been completely delaminated from the copper foil.

**References**

[1] Y. Wang, Y. Zheng, X. Xu, E. Dubuisson, Q. Bao, J. Lu, and K. P. Loh Electrochemical Delamination of CVD Grown Graphene Film: Toward the Recyclable Use of Copper Catalyst ACS NANO 5(2011)9927-9933
